# Health chatbots acceptability moderated by perceived stigma and severity: A cross-sectional survey

**DOI:** 10.1177/20552076211063012

**Published:** 2021-12-08

**Authors:** Oliver Miles, Robert West, Tom Nadarzynski

**Affiliations:** 1 Health Behaviour Research Centre, 4919 University College London Epidemiology and Public Health, London, UK; 2 School of Social Sciences, 4921 The University of Westminster, Westminster, UK

**Keywords:** Artificial intelligence, chatbots, bot, healthcare, acceptability, health bot

## Abstract

**Background:**

Chatbots and virtual voice assistants are increasingly common in primary care without sufficient evidence for their feasibility and effectiveness. We aimed to assess how perceived stigma and severity of various health issues are associated with the acceptability for three sources of health information and consultation: an automated chatbot, a General Practitioner (GP), or a combination of both.

**Methods:**

Between May and June 2019, we conducted an online study, advertised via Facebook, for UK citizens. It was a factorial simulation experiment with three within-subject factors (perceived health issue stigma, severity, and consultation source) and six between-subject covariates. Acceptability rating for each consultation source was the dependant variable. A single mixed-model ANOVA was performed.

**Results:**

Amongst 237 participants (65% aged over 45 years old, 73% women), GP consultations were seen as most acceptable, followed by GP-chatbot service. Chatbots were seen least acceptable as a consultation source for severe health issues, while the acceptability was significantly higher for stigmatised health issues. No associations between participants’ characteristics and acceptability were found.

**Conclusions:**

Although healthcare professionals are perceived as the most desired sources of health information, chatbots may be useful for sensitive health issues in which disclosure of personal information is challenging. However, chatbots are less acceptable for health issues of higher severity and should not be recommended for use within that context. Policymakers and digital service designers need to recognise the limitations of health chatbots. Future research should establish a set of health topics most suitable for chatbot-led interventions and primary healthcare services.

## Introduction

Primary healthcare is adopting alternative modes of delivery to address the growing demand for high-quality services, transitioning towards telemedicine and behaviour modification.^1^ This shift has been accelerated by the COVID-19 pandemic and subsequent social distancing measures facilitating digital innovation to shield vulnerable patients from being exposed to the infection.^2–3^ Conversational agents, chatbots or virtual assistants, are a type of digital technology underpinned by artificial intelligence (AI) or machine learning, designed to simulate human-to-human conversation via text or speech, able to understand health queries and provide a specific response in a conversational manner.^4–5^ One example is the Babylon “GP at Hand” service which incorporates chatbot functionality to triage patient’s health issues and direct them to relevant modes of care.^6^ The comparison of the diagnoses by doctors and the ‘GP at Hand’ service showed that the chatbot was able to diagnose the conditions with similar accuracy to human doctors.^7^ Thus, chatbots have the ability to communicate health information to patients aiding the less complex task within primary care.

Research into the acceptability of chatbots as consultation source is limited, with a distinct lack of experimental studies. Similar technologies, such as symptom checkers have been found to be acceptable to patients and staff in the primary healthcare context.^8^ Service users are willing to accept chatbots for disease diagnosis, given that they were not substituted for existing healthcare services.^9–10^ Chatbot acceptability is influenced by a person’s effort expectancy of using a chatbot, facilitating conditions, social influences, price value, habit, compatibility and perceived access to the healthcare system.^10^ The quality of the chatbot content, the perceived accuracy of health information, and the sources underpinning the chatbot are associated with acceptability. Their acceptability is low due to perceived chatbot responsibility, liability,^11–13^ perceived chatbot competence.^5,10,11^ Patient safety has also been identified as an important factor associated with acceptability.^11^ Low acceptability of chatbots, despite proven effectiveness, would lead to suboptimal uptake of this intervention. However, research indicates that people are more willing to disclose sensitive health information to chatbots in comparison to health professionals.^14–15^ Chatbot acceptability might be higher for stigmatised health issues as they offer greater anonymity than a face-to-face GP consultation.^11^

There is a need to establish how this novel technology can be best utilised within healthcare settings. Fadhil (2018) suggested that a GP-chatbot combination may increase chatbots acceptability and facilitate better patient-doctor communication, however, this mode of healthcare delivery has not been previously examined.^16^ As such, we aimed to assess how perceived stigma and severity are associated with chatbot acceptability by comparing that with acceptability for chatbot-led, GP-led and GP-chatbot combined consultation sources. The finding would inform the development of chatbot-guided services in primary healthcare.

## Methods

### Design

This study was an online factorial experiment design which included three within-subject factors (the stigma and the severity of the health issue and the consultation source) and six between-subject covariates (prior chatbot knowledge, confidence of chatbot knowledge, average internet usage, age, gender and education level). The outcome variable was participant rated acceptability of each consultation source. The study protocol and analysis plan were pre-registered (https://osf.io/szgma/). Approval was granted by the ethics committee of University College London (ref:14917/001).

### Participants and recruitment

The population of interest was any adult who might at some point have access to a chatbot for a health-related consultation. For cost and practical reasons, the study was conducted online using paid Facebook advertisement. All individuals aged 18 years and above with access to the internet, living in the UK were invited to participate. These inclusion criteria were reflected in the Facebook advertisement strategy, which cost £500 in total. Facebook users that fall into these criteria were shown with the study advert on their feed and were able to decide whether to take part in the study. It was unspecified how Facebook algorithm selected users to display its adverts and the researchers had little control over the sampling method. The target sample size was 250 participants, identified as an appropriate range for sufficient statistical power. No formal power calculations were made as the effect size was unknown.

Between May 2019 to June 2019, Facebook users were shown a digital advert inviting them to complete an online survey by clicking on an URL link, with an incentive of a 10 pence donation to a charity upon study completion. The advert asked if people could spare 10 min to fill out an online survey about attitudes towards the use of AI in healthcare. Once participants had accessed the online survey, they were presented with the information page and asked to consent.

### Measurements

In total, the survey consisted of seven items. First, participants were asked about the knowledge of chatbots. They were then presented with a video and text transcript describing the chatbot technology and its use in healthcare services and asked to rate their confidence in understanding the concept of a health chatbot. Later, they were presented with the 12 health issues such as ‘*you have been feeling severely depressed, and having suicidal thoughts*’, ‘*you have what you think are headlice’* and ‘*you have been coughing up blood’* (see supplementary file A) and asked to indicate the most preferred and acceptable consultation source. In the end, participants were asked demographic questions such as age, gender, educational attainment and internet usage.^17–18^

The outcome measure, acceptability of each consultation source, was based on the Acceptability Framework^19^ and was operationalised as a proxy measure ‘willingness’. Participants were presented with the statement *‘I would be willing to use this option to find out what is wrong and recommend treatment’* and were asked to rate their willingness to use each of the three consultation sources on a 5-point Likert scale (ranging from 1 = ‘not very willing at all’; to 5 = ‘very willing’) for each presented health issue. Scores under three were interpreted as a less acceptable rating by participants, while scores over three were interpreted as a more acceptable rating by participants.

The three experimental factors were: health issue stigma (more/less), health issue severity (high/low) and iii) consultation source (chatbot, GP, GP-chatbot combination). This resulted in 12 conditions (see supplementary file B).

The participants were randomly presented with three health issues for each more/less stigmatised or high/low severity condition to balance participant fatigue and practice effects. Each participant was asked to rate their acceptability of each of the three consultation sources for each consultation source. Participants were asked to complete 36 acceptability ratings and were blinded to the predicted stigma or severity of the health issue. This was to ensure that the participants retained their interpretation of the stigma and severity of the health issue.

### Data analyses

The means (*M*) and standard errors (*SE*) of acceptability ratings for chatbot, GP and GP-chatbot combination were calculated for all categories of participants where comparisons were being made. Both research questions were addressed together in a single mixed-model analysis of variance. The model included all main effects and all 2-way interactions involving consultation source. It also included the 3-way interaction between apparent stigma, severity and consultation source.

## Results

A total of 237 participants completed the study (Table 1). The majority were female (73.4%), aged over 45 years old (65.0%), and educated with a degree or higher (54.9%). Most had no prior knowledge of chatbots (59.5%) but were confident in their understanding of the technology once the concept was explained (70.9%).

**Table 1.** Sample characteristics and rated acceptability of chatbots.

Participant characteristic	Total of the sample (*%*)	Mean Acceptability Rating (SEM)
Gender		
Male	51 (*21.5*)	3.61 (*0.12*)
Female	174 (*73.4*)	3.38 (*0.08*)
Missing	12 (*5.1*)	
Age		
18–44	76 (*32.1*)	3.49 (*0.12*)
45+	154 (*65*)	3.50 (*0.09*)
Missing	7 (*3*)	
Educational level		
Anything else	99 (*41.8*)	3.38 (*0.10*)
Degree and above	130 (*54.9*)	3.60 (*0.09*)
Missing	8 (*3.4*)	
Prior knowledge		
No	141 (*59.5*)	3.57 (*0.10*)
Yes	75 (*31.6*)	3.41 (*0.11*)
Missing	21 (*8.9*)	
Confidence in chatbot knowledge		
Low confidence	68 (*29.1*)	3.42 (*0.13*)
High confidence	168 (*70.9*)	3.56 (*0.74*)
Missing	0 (*0*)	
Internet use		
Low usage	111 (*46.8*)	3.54 (*0.10*)
High usage	118 (*49.8*)	3.44 (*0.09*)
Missing	8 (*3.4*)	

*Note*: SEM = Standard Error of the Mean.

A significant main effect for consultation source (Table 2) *F*(2, 372) = 33.85, *p* < .001, partial *η*^2^ = 0.15 was found. GPs were reported as the most acceptable consultation source (*M* = 3.96, *SE* = 0.08), followed by a GP-chatbot combination (*M* = 3.43, *SE* = 0.11) and chatbots alone (*M* = 3.08, *SE* = 0.11). There was a significant interaction between acceptability and severity *F*(2, 372) = 118.14, *p* < .001, partial*η^2^* = 0.38, with GPs (*M* = 4.42, *SE* = 0.06), and a GP-chatbot combination (*M* = 3.44, *SE* = 0.12) being most acceptable for high severity health issues while chatbots least acceptable (*M* = 2.68, *SE* = 0.12). For low severity health issues, GPs were most acceptable (*M* = 3.51, *SE* = 0.11), followed by chatbots alone (*M* = 3.48, *SE* = 0.12), and GP-chatbot combination (*M* = 3.43, *SE* = 0.11). There was a significant interaction between acceptability and stigma *F*(2, 372) = 12.99, *p* < .001, partial *η* = 0.65, with GPs (*M* = 3.85, *SE* = 0.08), being most acceptable for high stigma health issues while a GP-chatbot combination (*M* = 3.42, *SE* = 0.11), chatbots alone (*M* = 3.14, *SE* = 0.12) showed moderate acceptability. For low stigma health issues, GPs (*M* = 4.08, *SE* = 0.08) were reported as the most acceptable consultation source, followed by the GP-chatbot combination (*M* = 3.45, *SE* = 0.12), and a chatbot alone (*M* = 3.02, *SE* = 0.11).

**Table 2.** The interactions between chatbot acceptability, perceived symptom severity and stigma.

Predictor	Health issue	Consultation source	Mean acceptability Rating (SEM)	F(df)	*p*-value
Consultation source		GP	3.96 (0.08)	33.85(2, 372)	<0.001
		GP-Chatbot	3.43 (0.11)		
		Chatbot	3.08 (0.110		
Perceived severity by Consultation source (interaction)	High severity	GP	4.42 (0.06)	188.14 (2, 372)	<0.001
		GP-Chatbot	3.44 (0.12)		
		Chatbot	2.68 (0.12)		
	Low severity	GP	3.51 (0.11)		
		GP-Chatbot	3.43 (0.11)		
		Chatbot	3.48 (0.12)		
Perceived stigma by Consultation source (interaction)	High stigma	GP	3.85 (0.08)	12.99 (2, 372)	<0.001
		GP-Chatbot	3.42 (0.11)		
		Chatbot	3.14 (0.12)		
	Low stigma	GP	4.08 (0.08)		
		GP-Chatbot	3.45 (0.12)		
		Chatbot	3.02 (0.11)		
Severity by Stigma by Consultation source (interaction)	High severity & High stigma	GP	4.20 (0.08)	16.47 (2, 372)	<0.001
		GP-Chatbot	3.44 (0.12)		
		Chatbot	2.85 (0.13)		
	High severity & Low stigma	GP	4.65 (0.05)		
		GP-Chatbot	3.43 (0.13)		
		Chatbot	2.51 (0.13)		
	Low severity & High stigma	GP	3.51 (0.11)		
		GP-Chatbot	3.41 (0.12		
		Chatbot	3.44 (0.13)		
	Low severity & Low stigma	GP	3.51 (0.13)		
		GP-Chatbot	3.46 (0.13)		
		Chatbot	3.52 (0.13)		

*Note*: SEM = Standard Error of the Mean, *df* = degrees of freedom.

There was a significant interaction between stigma, severity of the health issue and the acceptability of the consultation sources (Table 2) *F*(2, 372) = 16.47, *p* < .001, partial *η*^2^ = 0.081. GPs (*M* = 4.20, *SE* = 0.08) and GP-chatbot combination (*M* = 3.44, *SE* = 0.12) were found to be acceptable consultation sources for high stigma/high severity health issues, while chatbots (*M* = 2.85, *SE* = 0.13) were perceived as unacceptable consultation sources. For low stigma/high severity health issues, GPs (*M* = 4.65, *SE* = 0.05) and GP-chatbot combination (*M* = 3.43, *SE* = 0.13) were seen acceptable, while chatbots (*M* = 2.51, *SE* = 0.13) were perceived as unacceptable consultation sources. For high stigma/low severity health issues, GPs (*M* = 3.51, *SE* = 0.11), chatbots (*M* = 3.44, *SE* = 0.13), and a GP-chatbot combination (*M* = 3.41, *SE* = 0.12) were comparably seen as acceptable consultation sources. For low stigma/low severity health issues, chatbots alone were rated as the most acceptable consultation source (*M* = 3.52, *SE* = 0.13), followed by GPs (*M* = 3.51, *SE* = 0.13), and a GP-chatbot combination (*M* = 3.46, *SE* = 0.13).

With the between-subject factors, there was insufficient evidence to conclude that any of the factors influenced the acceptability ratings by participants (Table 1). There were non-significant main effects for: age *F*(1, 186) = 0.002, *p* = 0.97, partial *η*^2^ = 0.000; gender *F*(1, 186) = 2.97, *p* = 0.09, partial *η*^2^ = 0.016; educational level *F*(1, 186) = 3.64, *p* = 0.06, partial *η*^2^ = 0.019; prior chatbot experience *F*(1, 186) = 1.35, *p* = 0.25, partial *η*^2^ = 0.007; confidence in chatbot understanding *F*(1, 186) = 1.07, *p* = 0.3, partial *η*^2^ = 0.006; and time spent on the internet *F*(1, 186) = 0.82, *p* = 0.37, partial *η*^2^ = 0.004.

## Discussion

As far as we know, this is the first study to assess the associations between health chatbot acceptability and perceived stigma/severity of various health conditions. It demonstrated that in comparison to chatbots participants perceived health professionals as the most suitable source of health information. Chatbots were perceived as an unacceptable intervention for discussion health conditions that were perceived as severe. Therefore, this technology as a stand-alone intervention may not be utilised by healthcare customers and should not be used as a substitute for creditable health information source from a health professional. However, chatbots could be considered as an aid for doctor-patient communication for conditions with lower perceived stigma and severity. Future research needs to define the range of these conditions and identify how chatbots may facilitate greater disclosure of sensitive information to health professionals who may then be better equipped to recommend the most relevant healthcare services.

### Strengths and limitations

The study had several limitations. As it was advertised online via popular social media, the participants were more likely to be digitally literate and have access to technologies that would enable them to use chatbots. Thus, the views explored in the study may not reflect individuals with limited access to technology. The sample was highly skewed towards middle-aged female participants, which may reflect the majority of Facebook users in the UK at that time. Although Facebook advertising is considered a cost-effective and swift method for study recruitments, the participants are self-referred and may already hold strong views on the researched subject compromising the representativeness and applicability of the findings. The was also a significant percentage of missing data having an impact on the validity of the study. It is not clear why some questions were left unanswered, but this could be due to participants having a poor understanding of chatbots and not willing to respond to highly hypothetical questions. Furthermore, ‘willingness to use chatbot’ may not be the most precise measure of acceptability having the poor ability to predict motivation towards this technology. Other measures such as intentions or perceived likelihood to use chatbots could produce different results. To increase the representativeness or validity of the findings, future studies should consider offering survey alongside a health chatbot used in primary care, so that users can interact with the technology and provide more meaningful responses about their potential uptake and engagement.

As a simulation study, the conditions participants undertook were hypothetical, and the willingness to use chatbots may be different if participants experienced these conditions. Perceptions of severity and stigma may vary, hence the chatbot acceptability could be dependent on health beliefs. Patient groups for specific health conditions may be more or less reluctant to using chatbots. For example, this technology could be appealing to young adults at risk of sexually transmitted infections where disclosure of intimate information is challenging. However, chatbots may not be suitable for those suffering from psychotic episodes who require support and human care. Although none of the demographic characteristics was associated with chatbot acceptability in our study, future research should explore whether there are specific patient groups receptive to receiving health education through this technology.

These results build on existing evidence on chatbot acceptability in healthcare and triangulate the findings of the existing qualitative research into healthcare chatbot acceptability.^9–10,20^ The lower chatbot acceptability could be due to the lack of familiarity with the technology and hesitancy towards AI due to poor understanding of automated healthcare services. Only 70% of participants were confident in their understanding of chatbots after being given informational material explaining the functions and uses of health chatbots, suggesting that the concept of a computer service mimicking human responses may be difficult to comprehend. Previous studies also identified moderate acceptability of health chatbots, indicating ‘AI hesitancy’ in the healthcare context.^10^ There is a common misunderstanding that AI drives the function of chatbots. Chatbots can be complex and driven by AI through natural language processing. However, most chatbots are rule-based, i.e. if question A appears, response B should follow, thus more successful when the query is straightforward. Users may overestimate the capabilities of chatbots and be hesitant to use them due to the perception that chatbots could in the future replace healthcare professionals.

The lack of empathy from chatbots was seen as a major barrier to acceptability explaining the preferences for a human role in the type of health consultation. This was also reflected in higher acceptability for GP-chatbot combined consultation as opposed to chatbot alone demonstrating the potential use of this technology. It is also possible that lower chatbots acceptability, particularly in the UK, is associated with a lack of perceived need for such innovation a perception that chatbots could influence the access to health professionals.^12^ There is a general reluctance towards new technologies in healthcare, and future developments of chatbots and voice assistants may influence their acceptability and engagement.

Future theoretical development needs to consider how specific health issues influence the acceptability of chatbots for disease diagnosis. For example, the decision to accept a chatbot for diagnosis may be influenced by factors such as the urgency for diagnosis, the person’s wellbeing and how the individual feels about their symptoms. Future research should investigate whether user perceptions of usability differ according to countries and regions. Furthermore, it is vital to assess how various beliefs about particular diseases affect acceptability and engagement with medical chatbots. More comprehensive models of chatbot acceptability and engagement in healthcare can facilitate the understanding of their usefulness and applicability.

In conclusion, at present chatbots are not perceived as a desirable health intervention, and more research is needed to identify the level of interaction that is most acceptable to patients. Primary care services could consider chatbots as a signposting tool aiding health professional or improving doctor-patient communication for low severity conditions. Future studies need to explore whether chatbots could facilitate disclosure of sensitive information within the healthcare context; however, perceived confidentiality, privacy and security of the technology need to be ensured. Chatbot-led intervention and service developers need to be aware of moderate acceptability of this technology, taking into account digital literacy and access for the users.

## Supplemental Material

sj-docx-1-dhj-10.1177_20552076211063012 - Supplemental material for Health chatbots acceptability moderated by perceived stigma and severity: A cross-sectional survey Click here for additional data file. Supplemental material, sj-docx-1-dhj-10.1177_20552076211063012 for Health chatbots acceptability moderated by perceived stigma and severity: A cross-sectional survey by Oliver Miles, Robert West and Tom Nadarzynski in Digital Health

## References

[1] National Health Service. Long Term Plan. 2019. Retrieved from https://www.longtermplan.nhs.uk/ (2019; accessed 14 June 2019).

[2] Ohannessian R Duong TA Odone A . Global telemedicine implementation and integration within health systems to fight the COVID-19 pandemic: a call to action. JMIR public health and surveillance, 2020; 6, e18810. doi:10.2196/18810. 32238336 PMC7124951

[3] Robbins T Hudson S Ray P , et al. COVID-19: a new digital Dawn? Digital Health, 2020; 6. doi:10.1177/2055207620920083. PMC7153182 32313668

[4] Yu K-H Beam AL Kohane IS . Artificial intelligence in healthcare. Nature Biomedical Engineering 2018; 2: 719–731. doi:10.1038/s41551-018-0305-z. 31015651

[5] Palanica A Flaschner P Thommandram A , et al. Physicians’ perceptions of chatbots in health care: cross-sectional Web-based survey. J Med Internet Res 2019; 21: e12887. doi:10.2196/12887. 30950796 PMC6473203

[6] Armstrong S . The apps attempting to transfer NHS 111 online. Br Med J 2018; k156. doi:10.1136/bmj.k156. 29335297

[7] Razzaki S Baker A Perov Y , et al. (2018). A comparative study of artificial intelligence and human doctors for the purpose of triage and diagnosis. arXiv. doi:1806.10698. 10.3389/frai.2020.543405 PMC7861270 33733203

[8] Carter M Fletcher E Sansom A , et al. (2018). Feasibility, acceptability and effectiveness of an online alternative to face-to-face consultation in general practice: a mixed-methods study of webGP in six devon practices. BMJ open, 8, e018688. doi:10.1136/bmjopen-2017-018688. PMC5829586 29449293

[9] Laumer S, Maier C and Gubler FT (2019). Chatbot Acceptance in Healthcare: Explaining User Adoption of Conversational Agents for Disease Diagnosis. Proceedings of the 27th European Conference on Information Systems (ECIS).

[10] Nadarzynski T Miles O Cowie A , et al. Acceptability of artificial intelligence (AI)-led chatbot services in healthcare: a mixed-methods study. Digital Health 2019; doi:10.1177/2055207619871808. PMC6704417 31467682

[11] Luxton D. D. (Ed.). (2016). Artificial intelligence in behavioral and mental health care. Amsterdam: Boston: Elsevier/Academic Press.

[12] Meskó B . The role of artificial intelligence in precision medicine. Expert Review of Precision Medicine and Drug Development 2017; 2: 239–241. doi:10.1080/23808993.2017.1380516.

[13] Vaidyam AN Wisniewski H Halamka JD , et al. Chatbots and conversational agents in mental health: a review of the psychiatric landscape. The Canadian Journal of Psychiatry 2019; 4: 456–464. 070674371982897. doi:10.1177/0706743719828977. PMC6610568 30897957

[14] Lucas GM Gratch J King A , et al. It’s only a computer: virtual humans increase willingness to disclose. Comput Human Behav 2014; 37: 94–100.

[15] Lucas GM Rizzo A Gratch J , et al. Reporting mental health symptoms: breaking down barriers to care with virtual human interviewers. Frontiers in Robotics and AI 2017; 4: 51.

[16] Fadhil A . A Conversational Interface to Improve Medication Adherence: Towards AI Support in Patient’s Treatment. *ArXiv* 1803.09844 [*Cs]*. 2018; Retrieved from http://arxiv.org/abs/1803.09844.

[17] Government Statistical Service. Education attainment and qualifications. 2004. Retrieved from https://gss.civilservice.gov.uk/guidances/harmonisation/0-harmonised-principles/education-attainment-and-qualifications/#annex-a.

[18] Government Statistical Service. Harmonised concepts and questions for social data sources. 2017. Retrieved from https://gss.civilservice.gov.uk/wp-content/uploads/2016/03/Demographic-Info-June-17-Pending-informing-SPSC-1.pdf.

[19] Sekhon M Cartwright M Francis JJ . Acceptability of healthcare interventions: an overview of reviews and development of a theoretical framework. BMC Health Serv Res 2017; 17: 88. doi:10.1186/s12913-017-2031-8. 28126032 PMC5267473

[20] Grolleman J van Dijk B Nijholt A , et al. (2006). Break the habit! designing an e-therapy intervention using a virtual coach in Aid of smoking cessation. In: IJsselsteijn W. A. de Kort Y. A. W. Midden C. Eggen B. van den Hoven E. (Eds.), Persuasive technology (Vol. 3962, pp. 133–141). Springer Science & Business Media. Berlin, Germany. doi:10.1007/11755494_19.

