# Supplementary material for: Health chatbots acceptability moderated by perceived stigma and severity: A cross-sectional survey
Source: Digit Health. 2021 Dec 8;7:20552076211063012. doi: 10.1177/20552076211063012 (PMC8670785; doi:10.1177/20552076211063012)
Supplement: sj-docx-1-dhj-10.1177_20552076211063012 - Supplemental material for Health chatbots acceptability moderated by perceived stigma and severity: A cross-sectional survey [file sj-docx-1-dhj-10.1177_20552076211063012.docx]

**Supplementary Table 1**

**Perceived stigma and severity of health condition included in the stimulation**

| **Health Issue** | **Stigma** | **Severity** |
| --- | --- | --- |
| You have been feeling severely depressed, and having suicidal thoughts | High | High |
| You have not been able to defecate (pass stools) for more than 2 weeks | High | High |
| You have an abnormal genital discharge | High | High |
| You have what you think are headlice | High | Low |
| You have acne | High | Low |
| You have noticed a decrease in your libido | High | Low |
| You have been coughing up blood | Low | High |
| You have severe chest pain, nausea and breathlessness | Low | High |
| You have been finding breathing much more difficult | Low | High |
| You have had a mild cough for over 1 week | Low | Low |
| You have had a sore throat for a week, but no other symptoms | Low | Low |
| You have had an earache for 2 days, but no other symptoms | Low | Low |

**Survey measurements**

**Have you used a chatbot before?**

- Yes
- No
- Unsure

**Introduction video**

- A video outlining what a chatbot is using a screen video of someone using it (**script**)
  - Chatbots are natural language processing systems that act as a virtual conversational agent mimicking human interactions
  - Chatbots can provide you with instant responses for your health-related enquiries by looking for specific patterns of symptoms in predicting disease which can be tailored to specific populations, health conditions or behaviours.
  - The Chatbot plus GP approach for diagnosis involves using a chatbot to triage the user and direct them to a GP if needed, whose diagnosis will then be moderated by the chatbot.

**Do you feel confident that you know what a chatbot is?**

5-point Likert Scale

- Not very confident at all
- Very confident

**What we will ask you to do in this study**

You will be presented with a series of 12 hypothetical health scenarios. Your task is to imagine that you have the symptoms described and then say how willing you would be to use a chatbot, a clinician or a combination of the two. This should take no more than 5 minutes.

**The study**

*‘You have not been able to defecate (pass stools) for more than 2 weeks’*

I would be willing to use this option to find out what is wrong and recommend treatment

Chatbot

- Likert scale 1-5
  - Not very willing at all to very willing

I would be willing to use this option to find out what is wrong and recommend treatment

GP

- Likert scale 1-5
  - Not very willing at all to very willing

I would be willing to use this option to find out what is wrong and recommend treatment

Chatbot plus GP

- Likert scale 1-5
  - Not very willing at all to very willing

*‘You have been feeling severely depressed and having suicidal thoughts*

*‘You have abnormal genital discharge’*

*‘You have acne’*

*‘You have noticed a decrease in your libido’*

*‘You have what you think are headlice’*

*‘You have been coughing up blood’*

*‘You have been finding breathing much more difficult’*

*‘You have severe chest pain, nausea and breathlessness’*

*‘You have had a sore throat for a week, but no other symptoms’*

*‘You have had an earache for 2 days, but no other symptoms’*

*‘You have had a mild cough for over 1 week’*

High severity/ high stigma Low severity/ High stigma High severity/ Low stigma Low severity/ Low stigma

**Internet Frequency and Internet Usage**

In the last 3 months, how many hours per week do you think you have spent on the internet?

- Less than 5 hours;
- Between 5 and 9 hours
- Between 10 and 19 hours
- Between 20 and 29 hours
- Between 30 and 39 hours
- 40 hours or more.

Please indicate what you are most likely to use the internet for:

Communication:

a. E-mailing

b. Using social networking sites (e.g. Twitter, Facebook)

c. Accessing to chat sites, blogs, newsgroups or online discussions

d. Interacting with health or education professionals

e. Telephoning/video calling (via webcam)

Access to Information:

f. Finding information about goods and services

g. Reading / downloading online newspapers / news magazines

h. Seeking health information (on injury, disease, nutrition etc.)

i. Downloading software (other than games), patches or upgrades

Leisure:

j. Listening for web radios, watching web television

k. Playing/streaming/downloading games, images, films or music

l. Playing networked games with other persons

Professional life:

m. Looking for a job or sending a job application

n. Participating in professional networks (e.g. Linkedin)

Learning:

o. Doing an online course (in any subject)

p. Consult wikis (e.g. Wikipedia) or any online source of

Creativity/User-Generated Content (UGC)

q. Managing personal homepage

r. Uploading self-created content on sharing websites (e.g YouTube)

s. Blogging (maintaining or adding content to a blog)

Other activities:

t. Internet banking

u. Selling of goods or services (e.g. on eBay)

v. Using services related to travel or travel related accommodation

**About you**

Please select the options that best describe you

- Age
  - 18-24
  - 25-34
  - 35-44
  - 45-54
  - 55-64
  - 65-74
  - 75+
- Gender
  - Male
  - Female
  - Prefer not to say
  - Other: Please specify
- Highest education level
  - No qualification
  - GCSE’s/ O Level or equivalent
  - A level or equivalent
  - Undergraduate
  - Postgraduate or higher
